# PCB169 exposure aggravated the development of non-alcoholic fatty liver in high-fat diet-induced male C57BL/6 mice

**DOI:** 10.3389/fnut.2024.1350146

**Published:** 2024-05-07

**Authors:** Yunli Wei, Guangxian Zhou, Guangzhou Lv, Wan Wei, Lunguelizabeth Shera, Hongying Lin, Jinjun Chen, Danju Kang

**Affiliations:** Department of Veterinary Medicine, College of Coastal Agriculture, Guangdong Ocean University, Zhanjiang, Guangdong, China

**Keywords:** nonalcoholic fatty liver disease, high fat diet, lipid metabolism, transcriptome, PCB169, mouse

## Abstract

Polychlorinated biphenyls (PCBs) are lipophilic environmental toxicants. Epidemiological studies have established a link between PCBs and both metabolic syndrome and nonalcoholic fatty liver disease (NAFLD). Multiple studies have reported that exposure to both PCB156 and PCB126 among the 12 dioxin-like PCBs leads to the development of NAFLD. However, studies to elucidate whether PCB169 induces the development of NAFLD by constructing *in vivo* models have not been reported. Therefore, we evaluated the effects of exposure to PCB169 (5 mg/kg-bw) on hepatic lipid metabolism in C57BL/6 mice from control diet and high-fat diet cohorts. The results showed that PCB169 exposure reduced body weight and intraperitoneal fat mass in mice on the control diet, but the liver lipid levels were significantly increased, exacerbating NAFLD in mice on a high-fat diet. Through transcriptomics studies, it was found that PCB169 exposure induced significant up-regulation of *Ppar*γ, *Fasn*, and *Aacs* genes involved in hepatic lipogenesis, as well as remarkable up-regulation of *Hmgcr, Lss*, and *Sqle* genes involved in cholesterol synthesis. Additionally, there was notable down-regulation of *Ppar*α and *Cpt1* genes involved in lipid β-oxidation, leading to abnormal lipid accumulation in the liver. In addition, we found that PCB169 exposure significantly activated the Arachidonic acid metabolism, PPAR signaling pathway, Metabolism of xenobiotics by cytochrome P450, and Retinol metabolism pathways, and so on. Our study suggests that PCB169 can modify gene expression related to lipid metabolism, augument lipid accumulation in the liver, and further contribute to the development of NAFLD, thereby revealing the detrimental effects associated with PCB exposure on animal growth and metabolism.

## 1 Introduction

Non-alcoholic fatty liver disease (NAFLD) is a condition in which factors other than alcohol consumption contribute to the excessive fat deposition in hepatocytes, with a global prevalence of more than 25%, making it the world's number one chronic liver disease (1). NAFLD is a multifactorial disease often associated with metabolic disorders (2), such as diabetes (3) and hypertension (4). If left untreated, it can progress to more serious conditions such as cirrhosis and liver fibrosis (5), thereby affecting the health of animals and humans. Epidemiological studies have shown that exposure to environmental pollutants such as heavy metals, dioxins and PCBs can lead to hepatic steatosis (6), a condition closely linked to metabolic diseases and a potential factor contributing to NAFLD (7). This poses a significant threat to the health of both humans and animals. Amongst these environmental pollutants, polychlorinated biphenyls (PCBs), which possess high lipophilicity and slow metabolism, have attracted attention (8). Consequently, they can accumulate in the liver and adipose tissues over a lifetime through the amplification of the food chain in a stepwise manner (9), hence, affecting the body's neurology, reproduction, development, immunity and metabolism, etc (10).

PCBs have been classified as metabolism disrupting chemicals. However, there are 209 distinct types of congeners, and their metabolic toxicity can vary significantly depending on their structure. Twelve coplanar PCBs are known as Dioxin-like Polychlorinated biphenyls (DLPCBs), which are similar to 2,3,7,8-tetrachlorodibenzo-p-dioxin (11, 12). Despite the fact that commercial production of PCBs has been banned in most countries since the 1970s (13), these compounds are still detected in animals, human blood and various environmental media (14, 15). PCBs in the environment mainly comes from lubricants, transformers, transformer vessels, heat exchangers and so on (16). Exposure to these compounds occurs primarily through diet (17). The concentration of PCB169 in the blood of Shenzhen residents is 13.7 ± 6.78, 15.2 ± 7.60, and 21.1 ± 11.2 pg/g lipid in the blood of those aged <30, 31–40, and 41–50, respectively. It can be seen that the blood concentration of PCB169 is positively correlated with age (18). PCB169 was also detected in pork, beef, fish and shrimp in Shenzhen, with concentrations ranging from 0.013 to 0.872pg/g ww (18). The main source of exposure to PCB169 is from eating contaminated animal foods. Studies have shown a dose-dependent relationship between the concentration of PCBs in the serum of animals and the risk of developing NAFLD. Other distinguishable studies have reported that PCB126 exposure to hepatocytes enhanced the content of intracellular lipid and the expression levels of SREBP1 and DGAT-2 proteins involved in lipid accumulation, hence promoting the development of NAFLD (19). PCB156 or PCB153 were exposed to mice, and found that the mice gained body weight, had an increase in intra-abdominal adiposity, and that the genes involved in hepatic lipogenesis, such as *PPAR*γ and *Fas*, were up-regulated. Furthermore, expression of the lipid β-oxidative catabolic genes such as *Cpt1b* and *PPAR*α were down-regulated, thereby promoting lipid accumulation (20, 21). In addition, PCB156 exposure notably activated the cytochrome P450 pathway, peroxisome proliferator-activated receptors (PPARs), and glutathione metabolic pathways, and significantly changed the expression of related genes. Specifically, PCB 156 exposure upregulated the expression of *CD36* to increase fatty acid influx and downregulated the expression of *Cpt1b* and *Acaa1b* to decrease lipid oxidation. Moreover, PCB 156 increased *PPAR*γ, *Osbol3*, and *Srebp1c* expression to promote FAs synthesis. Abnormal accumulation of lipids in the liver promotes the development of NAFLD. These studies suggest that exposure to PCBs increases the likelihood of NAFLD occurring (21). However, there have been a few *in vivo* modeling studies that have reported on the effects of PCB169 on NAFLD, and the exact molecular mechanisms remain unclear. Studies have revealed that nutrients have the potential to interact with toxicants and can further exacerbate hepatotoxicity and metabolism-related diseases (22). Therefore, we conducted an experiment where mice were exposed to PCB169 (5 mg/kg-bw) at different nutrient levels for 8 weeks to study the effect of PCB169 on hepatic lipid metabolism by observing liver pathological histology, determining the level of hepatic lipid content, and analyzing changes in lipid metabolism pathways and expression of lipid synthesis and catabolism genes at the level of transcriptomics, so as to understand how the toxicity of PCB169 is manifested at molecular level, and to assess the effect of nutrient and PCB169 co-exposure on NAFLD. This study therefore provides a new perspective into the toxic effects of PCB169 exposure on the liver and its mechanisms.

## 2 Materials and methods

### 2.1 Materials

PCB169 (CAS No. 32774-16-6, Purity > 98%) was purchased from Dr. Ehrenstorfer (Germany) and dissolved in corn oil (Biyuntian, Shanghai, China) prior to the experiment. The control diet comprised of 12.0% kCal from fat, while the high fat diet comprised of 42% kCal from fat (Synergistic Pharmaceuticals, Jiangsu, China). The detailed composition of the diets can be found in Supplementary Table S1. The reagents, such as 4% paraformaldehyde solution, anhydrous ethanol, etc., were domestically produced and analytically pure.

### 2.2 Animal studies

Forty SPF-grade male C57BL/6 mice (7 weeks old) weighing 18-22 g were purchased from Zhuhai Biotest Biotechnology Co. They were placed in a controlled environment with a temperature of 23±2°C, humidity of 55±10%, and a light/dark cycle of 12:12 h, and provided with *ad libitum* diet and water. After 1 week of acclimatization feeding, 40 mice were randomly divided into four experimental groups (10 per group): control diet (CL), CL + 5 mg/kg-bw PCB169 (CPL), and high-fat diet (HL), HL + 5 mg/kg-bw PCB169 (HPL). PCB169 (5 mg) was added to 5 mL of corn oil until completely dissolved, prepared every 2 weeks. During the 8-week experimental period, mice were treated weekly by gavage with either corn oil (CL, HL) or PCB169 dissolved in corn oil (CPL, HPL), and were weighed and measured once a week. The mice were given a dose of 5 mL/kg by oral gavage. The mice were treated with PCB169 once a week. This study was conducted to investigate the effects of low doses of PCB169 on lipid metabolism. Based on previous studies with PCBs, four intraperitoneal injections of PCB77 (49 mg/kg) during the 6-week study period showed increased serum cholesterol levels in mice (23). In a 12-week study, administration of 50 mg/kg PCB153 at different weeks, that is a total of 4 times resulted in abnormal increases in triglyceride and cholesterol levels. in the liver of mice, and PCB153 aggravated NAFLD in high-fat fed mice (20). Four intraperitoneal injections of 55 mg/kg PCB156 in mice resulted in an increase in intra-abdominal fat volume, abnormalities of hepatic lipid levels and lipids, and exacerbation of non-alcoholic fatty liver disease (NAFLD) in the PCB156 group following co-exposure to a high-fat diet and PCB156 (21). It has been reported that lower concentrations of PCBs may have the opposite effect on weight gain (5). In addition, mice in this study were exposed to PCB169 at 5 mg/kg-bw because of its high toxicity equivalency factor (24). PCBs have a half-life of several years, so the concentrations of PCBs in this study were lower than those previously reported to disrupt lipid metabolism.

The mice were euthanized with an injection of sodium pentobarbital (40 mg/kg-bw) after they had been starved for 12 h prior to dissection (20). The mice were sacrificed by cervical dislocation, the liver and adipose tissue were isolated, and weighed using electronic balance. Half of the collected liver tissues were frozen and stored in a −80°C refrigerator for subsequent experiments, and the rest was put in 4% paraformaldehyde. All animal experiments were conducted in accordance with the guidelines for the care and use of laboratory animals of Guangdong Ocean University, and approved by the Animal Ethics Committee of Guangdong Ocean University (Approval No. 2019090504) in an effort to minimize animal suffering.

### 2.3 Histological analysis

The liver tissues (*n* = 3/group) were fixed in 4% paraformaldehyde solution for 48 h and embedded in paraffin before being sectioned. The thickness of paraffin HE section was 3 μm, and that of frozen oil red section was 7 μm. The sections were stained with hematoxylin & eosin (HE) and oil red staining, and the sealed sections were processed for observation and analysis under a light microscope (SOPTOP, EX31).

### 2.4 Biochemical analysis

Liver samples were analyzed for total Cholesterol (TC), triglyceride (TG), low density lipoprotein Cholesterol (LDL-C), high density lipoprotein Cholesterol (HDL-C), aspartate transaminase (AST), and alanine transaminase (ALT) content using commercial kits according to the manufacturer's protocol (Nanjing Jiancheng Bioengineering Institute, China).

### 2.5 RNA extraction and quality assessment

Total RNA from the liver tissue of each mouse (*n* = 10) was extracted with TRIzol (Life technologies, California, USA). The purity and concentration of RNA were then detected by NanoDrop 2000 (Thermo Fisher Scientific, Wilmington, DE) spectrophotometer, and the integrity of RNA was detected by 1% agarose gel electrophoresis. Six RNA samples with high quality were selected for sequencing in each group.

### 2.6 cDNA library construction, RNA sequencing, and gene expression analysis

Random interruption and end repair were performed on the samples that passed the test, and thereafter the cDNA library was constructed by PCR enrichment. The quality of the library was accurately quantified by qPCR. After passing the quality control, it was sequenced using Illumina NovaSeq6000 platform PE150 (San Diego) mode.

Firstly, the low-quality raw data and reads containing joints were removed to get high quality data. Low-quality Reads included those with a ratio of N > 10% and a mass value of Q <10. The high-quality data were then sequence aligned with the reference genome of mice (GRCm39, Mus musculus) using HISAT2 (version 2.0.4) software (21). FPKM was used to reflect the expression of the transcript mRNA. Differentially expressed genes (DEGs) were screened using DESeq2 (version 1.30.0) software based on the gene Count value in each sample with Fold Change ≥ 2 and FDR <0.05 (25). Principal component analysis (PCA) was used to assess inter-group differences and sample duplication within groups.

### 2.7 GO and KEGG enrichment analyse of DEGs

Gene Ontology (GO) was employed to analyse the functions of DEGs. All the DEGs were matched to the GO terms in the database (http://www.geneontology.org/) and the gene numbers in each term were calculated. The significantly enriched GO terms for the DEGs were find. Kyoto Encylopedia of genes and genomes (KEGG) data base (http://www.genome.jp/kegg/) were was used for analyzing the functions of the DEGs in some biologic process. The KEGG pathways with *q* <0.05 were thought to be significantly enriched for the DEGs.

### 2.8 q-PCR validation

The genes of interest were picked according to the results of transcriptome analysis, and verified by real-time fluorescent quantitative PCR (q-PCR). The primer sequences are shown in Supplementary Table S2. The qPCR reaction procedure was performed as follows: initial pre-denaturation at 94°C for 30 s, followed by denaturation at 94°C for 5 s, annealing at 60°C for 15 s, and extension at 72°C for 10 s, with a total of 40 cycles. β-actin was selected as the internal reference gene, and the expression level of the gene was calculated using the 2^−Δ*ΔCt*^ method.

### 2.9 Data analysis

The experimental data in this study were statistically analyzed using GraphPad Prism version 8.0 (GraphPad), and the data results were expressed as mean ±SEM. The statistical significance was considered when the *P*-value was less than 0.05 (P <0.05). Normality test was performed by skewness coefficient and kurtosis coefficient using SPSS before conducting two-way ANOVA, and multiple comparisons were performed using Tukey test.

## 3 Results

### 3.1 PCB169 exposure increases hepatic lipid accumulation

**Figure 1:**
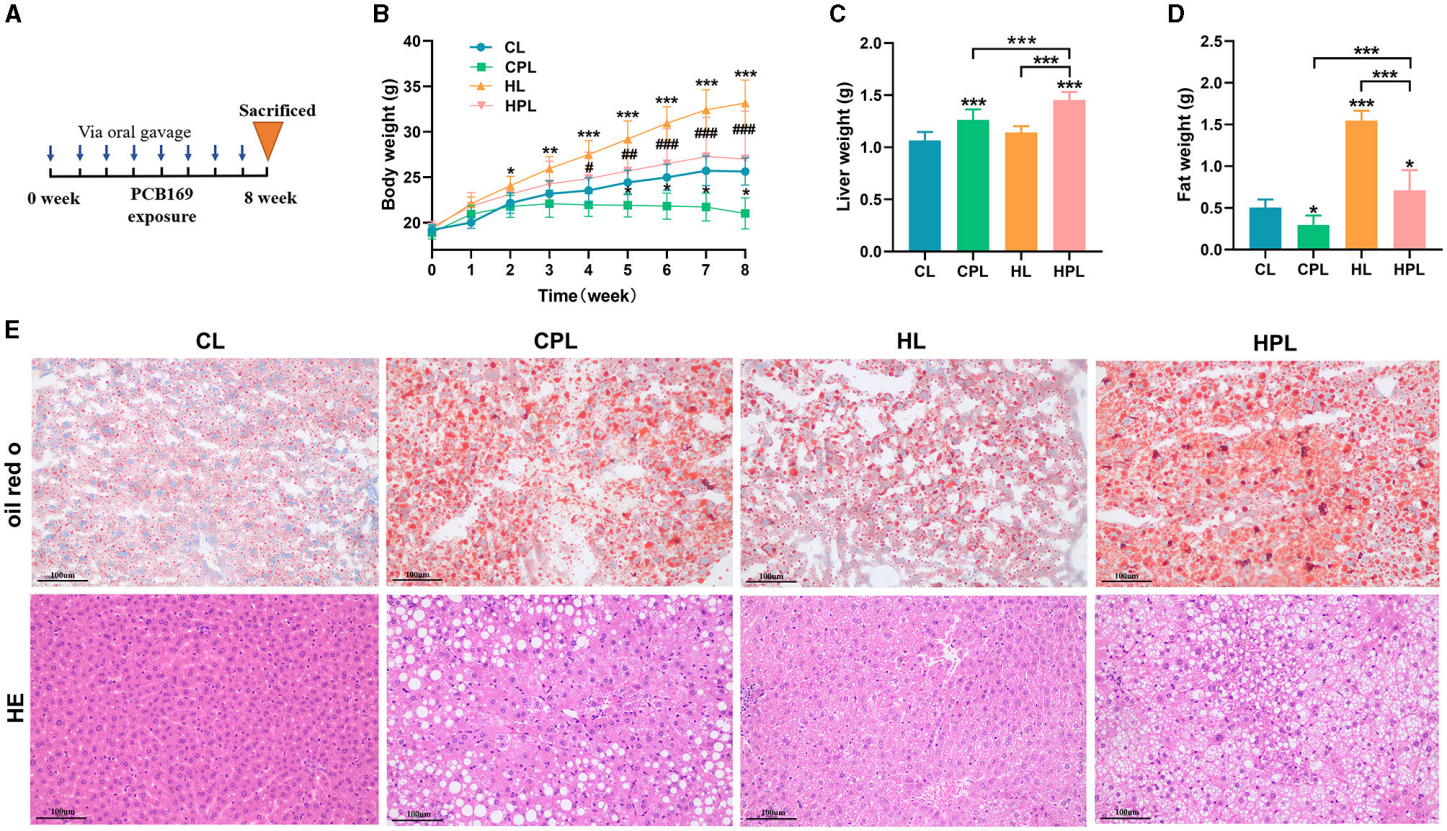
Effects of PCB169 exposure on body weight and liver weight of mice. **(A)** PCB169 exposure mode. **(B)** Changes in body weight of mice during the experimental period (*n* = 10). **(C)** Liver weight (*n* = 10). **(D)** Fat weight (*n* = 10). **(E)** Morphohistological changes in liver of mice. Oil red staining and HE staining (200 × ). * indicates *P < 0.05, **P < 0.01, ***P < 0.001 compared with CL group; ^#^ indicates ^#^*P* < 0.05, ^##^*P* < 0.01, ^###^*P* < 0.001 compared with HL group.

An illustration of PCB169 administration by oral gavage was shown in Figure 1A. After 8 weeks of exposure, the mice in the HL group had significantly higher body weight than the other groups. However, the average body weight of mice in the CPL group was significantly lower than that of the CL group by 22% (*P* < 0.001), and the average body weight of mice in the HPL group was significantly lower than that of the HL group by 22.7% (*P* < 0.001) (Figure 1B). Furthermore, we noticed a correlation between the variations in body weight among the four groups of mice and a decrease in intra-abdominal fat content. There was no statistically significant difference in average body weights between the mice in the HPL group and the CL group (Figure 1B). The liver weight showed a significant increase of 18.7% (*P* < 0.001) and 36.5% (*P* < 0.001) in the HL and HPL groups, respectively, compared with the CL group (Figure 1C). Furthermore, there was a significant increase of 29.3% (*P* < 0.01) in liver weight in the HPL group compared with the HL group (Figure 1C). In addition, the liver weight in the HPL group was notably increased by 15% (*P* < 0.001) compared to the CPL group (Figure 1C). The fat weight showed a significant reduce of 41.3% (*P* < 0.05) in the CPL groups, compared with the CL group (Figure 1D). Furthermore, there was a significant reduce of 54.1% (*P* < 0.001) in fat weight in the HPL group compared with the HL group (Figure 1D). In addition, the fat weight in the HPL group was notably increased by 1.39-fold (*P* < 0.001) compared to the CPL group (Figure 1D). Therefore, we further assessed the effect of PCB169 on the liver by performing liver histological analysis, as shown in Figure 1E, where oil red O staining indicated lipid accumulation in the livers of CPL-treated mice. The degree of lipid accumulation was more serious in mice in the HL and HPL groups compared with the CL group. The HE staining results in Figure 1E showed that the livers of mice in the CPL group showed lipid droplet vacuoles, and those in the HPL group showed a large number of lipid droplet vacuoles and inflammatory cell infiltration, compared with those in the CL group.

### 3.2 PCB169 alters liver biochemical indices

**Figure 2:**
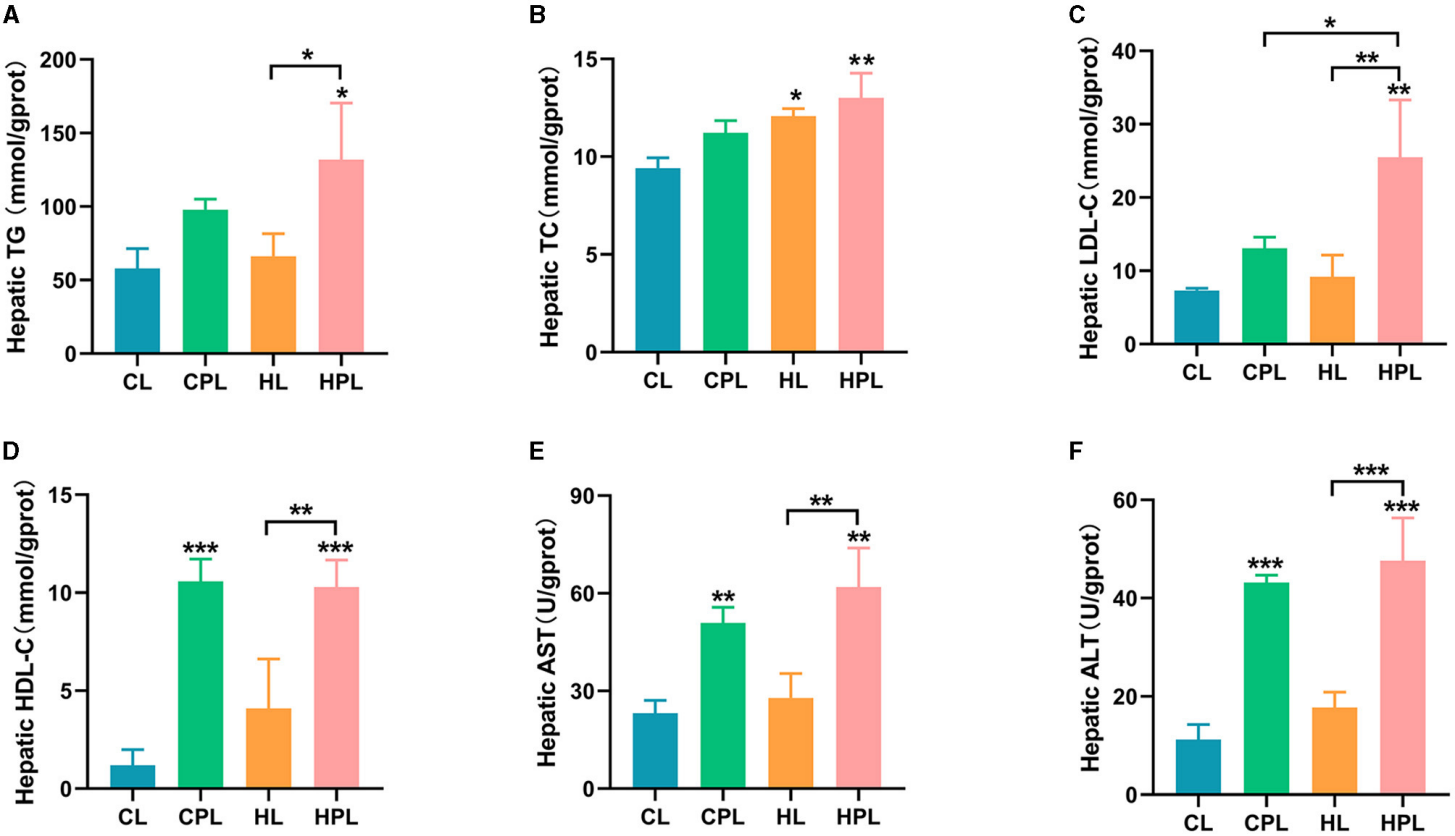
Effect of PCB169 exposure on liver biochemical indices. **(A)** Liver TG content. **(B)** Liver TC content. **(C)** Liver LDL-C content. **(D)** Liver HDL-C content. **(E)** Liver AST level. **(F)** Liver ALT level (*n* = 5). * indicates *P < 0.05, **P < 0.01, ***P < 0.001 compared with the CL group.

We next evaluated the biochemical index of the liver in each group of mice, which were hypothesized to be changed in liver histology induced by PCB169 exposure. CPL had no significant effect on hepatic TG, TC, and LDL-C content compared to the CL group, but the HPL group showed a considerable increase in TG (1.28-fold, *P* < 0.05), and highly significant increases in TC (0.38-fold, *P* < 0.01) and LDL-C (2.49-fold, *P* < 0.01) content (Figures 2A–C). Compared with the HL group, the HPL group induced 0.99-fold (*P* < 0.05), 0.08-fold, and 1.78-fold (*P* < 0.01) increases in hepatic TG, TC, and LDL-C contents, respectively (Figures 2A–C). In addition, LDL-C content increased 0.95-fold (*P* < 0.05) in the HPL group compared with the CPL group (Figure 2C). In comparison with the CL group, the hepatic HDL-C content was significantly increased by 7.8-fold (*P* < 0.001) and 7.6-fold (*P* < 0.001) in the CPL and HPL groups, respectively (Figure 2D). The HPL group showed a remarkable increase of 1.5-fold (*P* < 0.01) in HDL-C content compared with the HL group (Figure 2C). Additionally, hepatic TG, TC, LDL-C and HDL-C contents were elevated in mice in the HL group compared with the CL group. The above results indicate that PCB169 exposure can lead to abnormal accumulation of hepatic lipids in CL and HL fed mice.

In this study, common indicators including ALT and AST of liver injury were measured. As shown, hepatic AST and ALT levels were increased by 1.2-fold (*P* < 0.01) and 2.8-fold (*P* < 0.001) in the CPL group compared to the CL group (Figures 2C, D). Hepatic AST and ALT levels were increased by 1.7-fold (*P* < 0.01) and 3.2-fold (*P* < 0.001) in the HPL group compared to the CL group (Figures 2C, D). AST (1.2-fold, *P* < 0.01) and ALT (1.7-fold, *P* < 0.001) levels were significantly increased in the HPL group compared with the HL group (Figures 2C, D). The above results suggest that PCB169 induces liver injury in mice, especially in mice co-exposed to HL diet and PCB169.

### 3.3 PCB169 exposure alters the liver transcriptome

**Figure 3:**
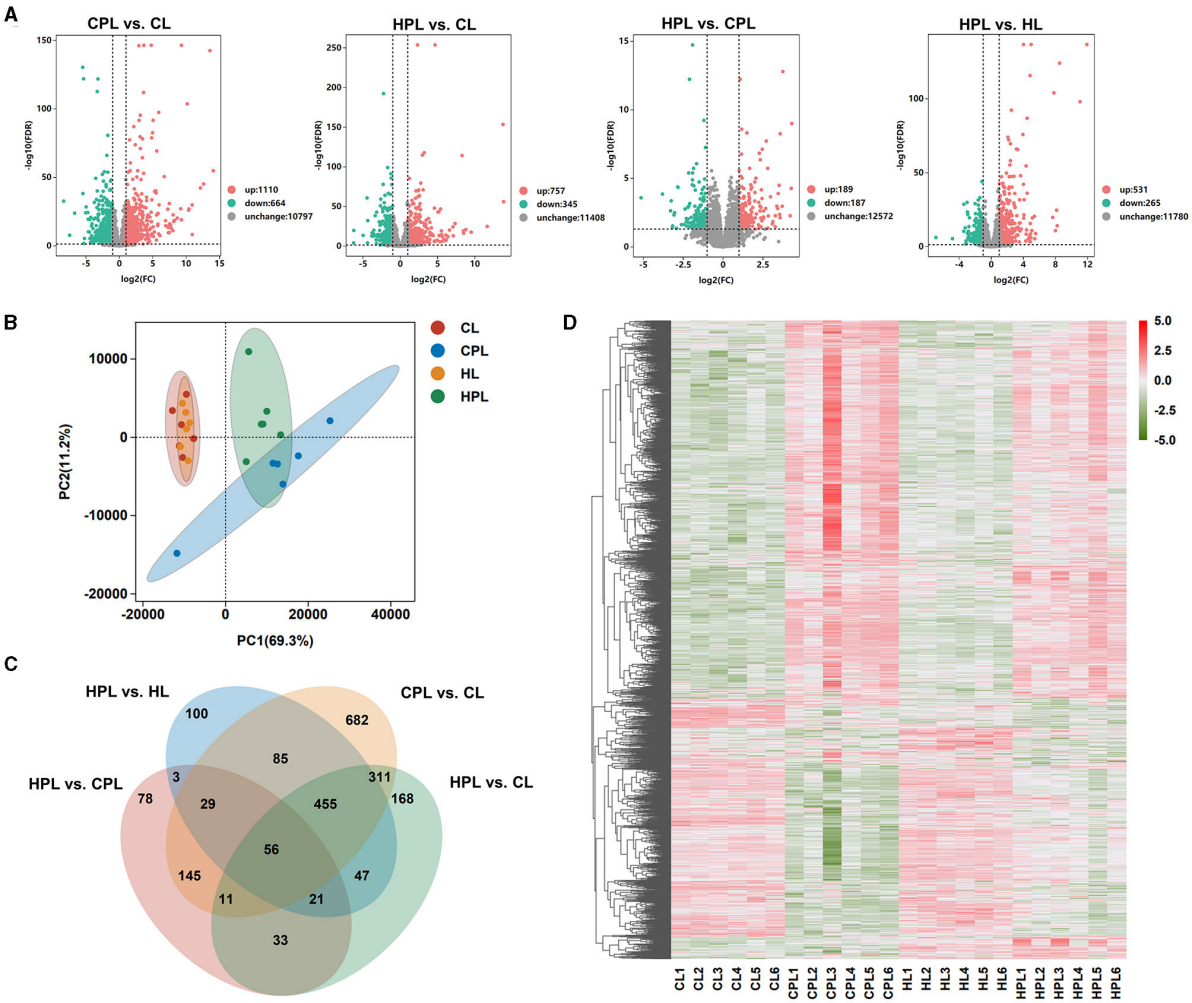
Transcriptome analysis of liver tissues exposed to PCB169. **(A)** Volcano plot of the distribution of DEGs. **(B)** PCA plot of transcriptome differences between samples. **(C)** Venn diagram showing DEGs expressed individually and co-expressed in the comparative groups. **(D)** Heatmap of DEGs in the 4 groups. Red color indicates genes up-regulated after PCB169 exposure whereas green color indicates genes down-regulated (*n* = 6).

In this study, to better understand the effects of PCB169 exposure on the liver and its related mechanisms through RNA-seq detailed data, a total of 24 cDNA libraries was established from 24 mouse liver samples (4 groups of 6 mice each) and analyzed using RNA-seq. DEGs were screened according to the multiplicity of differences and significant levels, and the overall distribution of DEGs in the four analyzed groups is shown in Figure 3A. Compared with the CL group, 1,174 genes (1110 up-regulated and 664 down-regulated) and 1,102 genes (757 up-regulated and 345 down-regulated) were significantly and differentially transcribed in the CPL and HPL groups, respectively (Figure 3A, Supplementary Table S3). 376 DEGs were found between the HPL and CPL groups, of which 189 genes were up-regulated and 187 genes were down-regulated. In addition to 796 DEGs of which 531 up-regulated and 265 down-regulated genes were found between the HPL and HL groups (Figure 3A, Supplementary Table S3). Principal component analysis (PCA) results showed that the PCB169 exposed group (CPL, HPL) and the unexposed group (CL, HL) differed significantly (Figure 3B). Special and common DEGs were next analyzed in the four comparison groups. As shown in Figure 3C, 682 DEGs were expressed only in the CPL group and 168 DEGs were expressed in the HPL group compared to the CL group. 78 and 100 DEGs were independently expressed in the HPL group compared to the CPL group and the HL group, respectively. In addition, 56 of the same DEGs were expressed in the four comparison groups (Figure 3C, Supplementary Table S4). Hierarchical clustering analysis was next performed for all DEGs (2,224 genes) in the above four comparison groups. In the gene expression clustering heatmap (Figure 3D, Supplementary Table S5), gene expression levels in the CPL and HPL groups were significantly different compared to the CL group. The HPL group showed notable differences in gene expression levels compared to the HL group. However, there was less change between PCB169-exposed groups. These results suggest that PCB169 exposure induces changes in the mouse liver transcriptome.

### 3.4 GO functional annotations of DEGs

**Figure 4:**
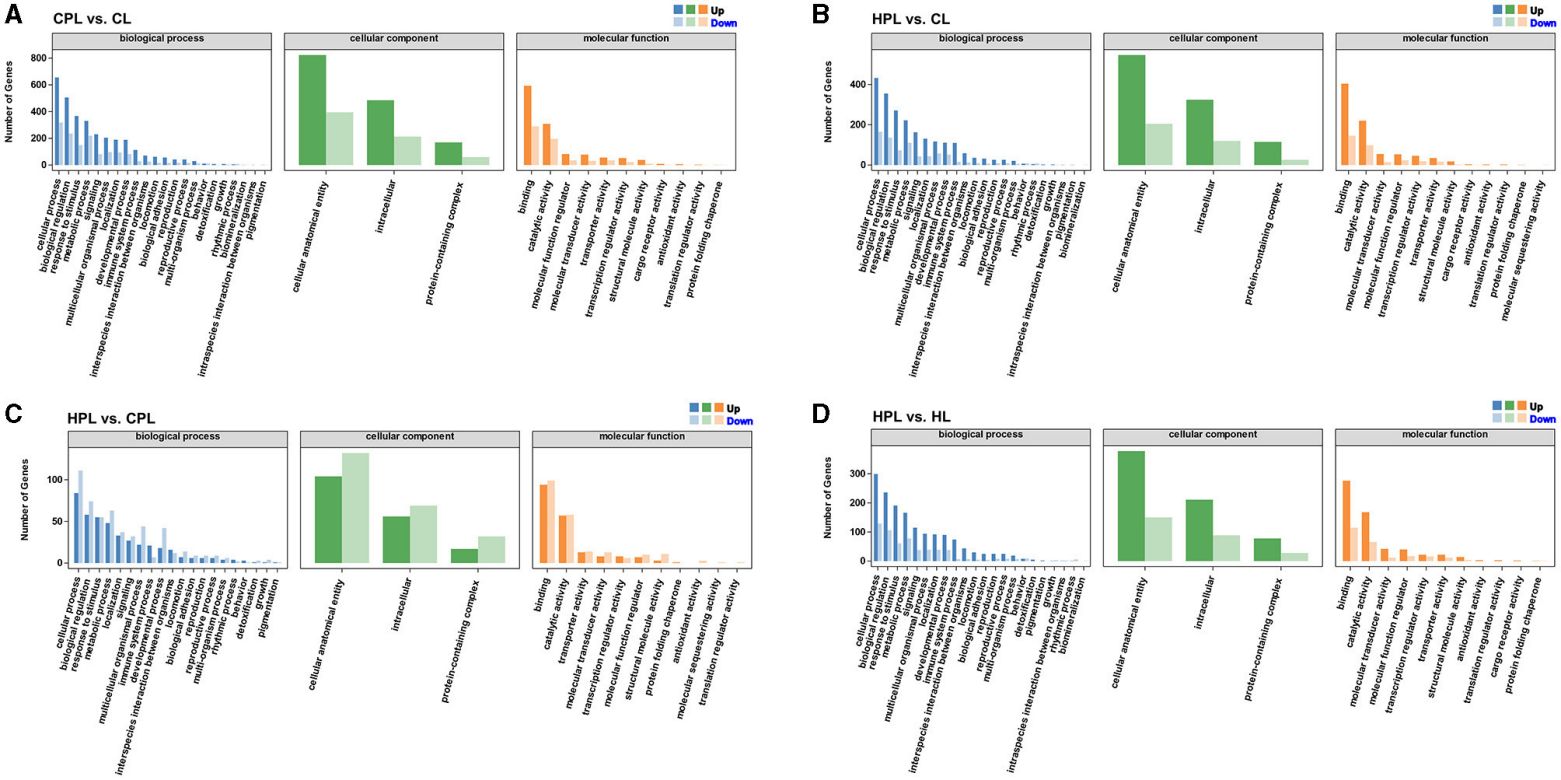
GO functional annotations analysis of DEGs. **(A)** CPL vs. CL. **(B)** HPL vs. CL. **(C)** HPL vs. CPL. **(D)** HPL vs. HL. x-coordinate denotes GO categorization; y-coordinate denotes the number of genes.

To better visualize the role of these DEGs in mouse liver, we performed GO functional annotations of DEGs in each comparison group, including biological processes, cellular components and molecular functions. The DEGs from these four comparison groups were enriched in similar GO Terms. Most of DEGs in the biological process category were connected to both cellular process, biological regulation, response to stimulus and metabolic process. In the cellular component category, most DEGs were assigned to the cellular anatomical entity, intracellular and protein-containing complex subcategories. While in another molecular function variety, most DEGs were classified as binding and catalytic activity (Figures 4A–D, Supplementary Table S6).

### 3.5 KEGG enrichment analysis of DEGs

**Figure 5:**
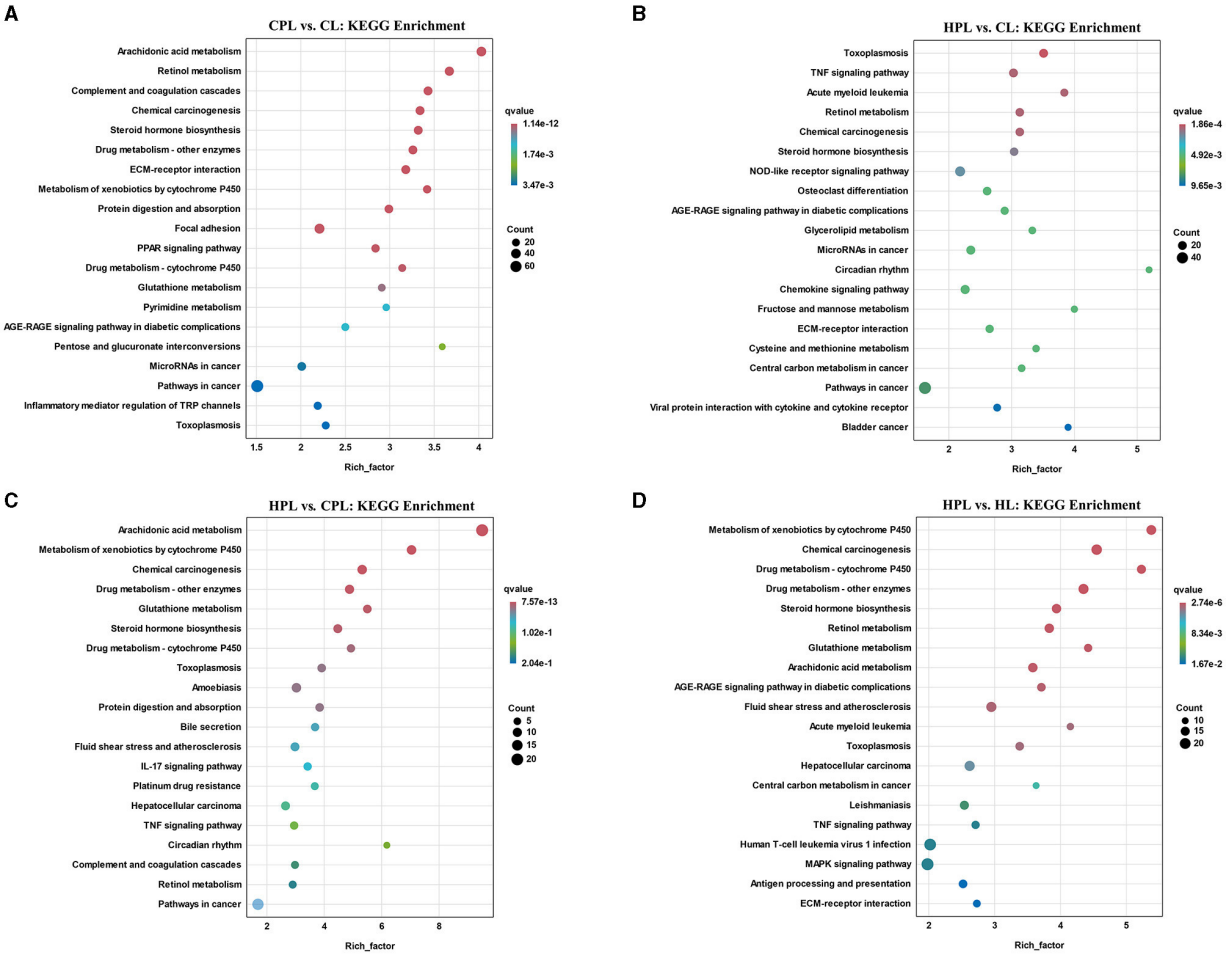
Top 20 KEGG pathways with the highest enrichment of DEGs. **(A)** CPL vs. CL. **(B)** HPL vs. CL. **(C)** HPL vs. CPL. **(D)** HPL vs. HL. X-coordinate denotes the enrichment factor of the genes. Y-coordinate denotes the pathway name, the size of the dots denotes the number of genes and the color of the dots denotes the size of the *q*-value.

Subsequently, we analyzed the top 20 KEGG pathways that exhibited the highest enrichment of DEGs in the four comparison groups. In the CPL vs. CL comparison, the most significantly enriched pathway was arachidonic acid metabolism (*q* = 1.14E-12), followed by retinol metabolism, complement and coagulation cascades, chemical carcinogenesis, and steroid hormone biosynthesis (Figure 5A, Supplementary Table S7). In the HPL vs. CL comparison, we found that several pathways, such as retinol metabolism, steroid hormone biosynthesis and pathways in cancer, were also enriched (Figure 5B, Supplementary Table S7). However, the most significantly enriched pathway in HPL vs. CPL was arachidonic acid metabolism (21 DEGs; *q* = 7.57E-13) (Figure 5C, Supplementary Table S7). In the HPL vs. HL comparison, the most significantly enriched pathway was metabolism of xenobiotics by cytochrome P450 (*q* = 2.74E-06) (Figure 5D, Supplementary Table S7).

### 3.6 PCB169 exposure promotes the expression of genes related to lipid synthesis in mouse liver

**Figure 6:**
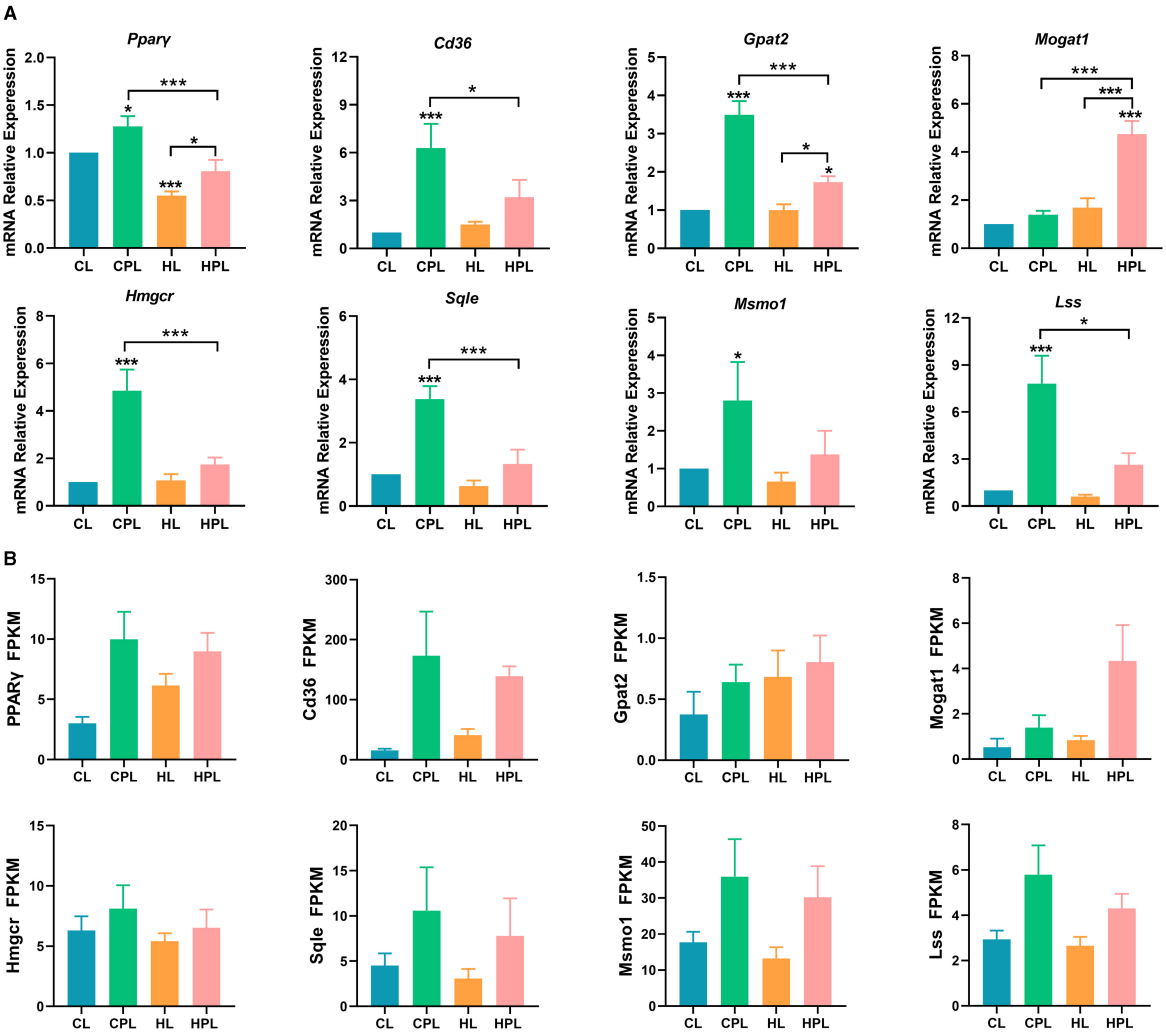
PCB169 exposure altered hepatic lipid metabolism-related gene expression. **(A)** Relative mRNA levels of lipid accumulation genes. **(B)** The overall trends of RAN-seq and qPCR results were basically the same. * indicates *P < 0.05, ***P < 0.001 compared with the CL group.

By analyzing the GO and KEGG enrichment results, we found that PCB169 exposure significantly affected lipid metabolism in mouse liver. To further investigate the effects of PCB169 on lipid metabolism, we analyzed the expression of key regulatory genes of mouse liver lipid metabolism induced by PCB169 exposure. The qPCR results are shown in the Figure 6. The expression of the genes *Ppar*γ, a gene related to lipid synthesis, and *Cd36*, a key gene for fatty acid transport, were significantly increased by 0.28-fold (*P* < 0.05) and 5.30-fold (*P* < 0.001), respectively, in the CPL group as compared with the CL group (Figure 6A). HPL showed a 0.47-fold (*P* < 0.001) and 1.13-fold increase in *Ppar*γ gene and *Cd36* gene expression, respectively, compared with the HL group (Figure 6A). In addition, *Ppar*γ gene and *Cd36* gene expression were significantly reduced by 36.8% (*P* < 0.001) and 49.2% (*P* < 0.05) in the HPL group compared with the CPL group, respectively (Figure 6A). The expression of *Gpat2* and *Mogat1* genes, which are directly involved in TG synthesis, was up-regulated to different degrees in the PCB169-exposed group compared with the CL group. The expression of *Gpat2* gene in the CPL group was significantly increased by 2.49-fold (*P* < 0.001) compared with the CL group (Figure 6A). The *Gpat2* gene and *Mogat1* gene exhibited a significant increase by 0.73-fold (*P* < 0.05) and 3.74-fold (*P* < 0.001) respectively in HPL compared with CL group (Figure 6A). In addition, the expression levels of both *Gpat2* and *Mogat1* genes in the HPL group differed significantly from those in to the CPL group. Additionally, the expression levels of the genes of the cholesterol synthesis pathway, *Hmgcr, Sqle, Lss* and *Msmol*, were significantly up-regulated in the CPL and HPL groups. Particularly, in the CPL group, there was a 3.85-fold (*P* < 0.001), 2.38-fold (*P* < 0.001), 6.82-fold (*P* < 0.001), and 1.80-fold (*P* < 0.05) increase in the CPL group, respectively, compared with the CL group (Figure 6A). *Hmgcr, Sqle, Lss*, and *Msmol* gene expression levels in the HPL group were reduced by 64.1% (*P* < 0.001), 60.7% (*P* < 0.001), 66.2% (*P* < 0.05), and 50.8% compared with the CPL group (Figure 6A). The qPCR results were generally consistent with the trend of RNA-seq data (Figure 6B). Our data suggest that PCB169 exposure may be affecting lipid metabolism in mouse liver through the expression of genes that promote lipid accumulation.

### 3.7 PCB169 exposure promotes hepatic lipogenesis and inhibits lipid degradation in mice

**Figure 7:**
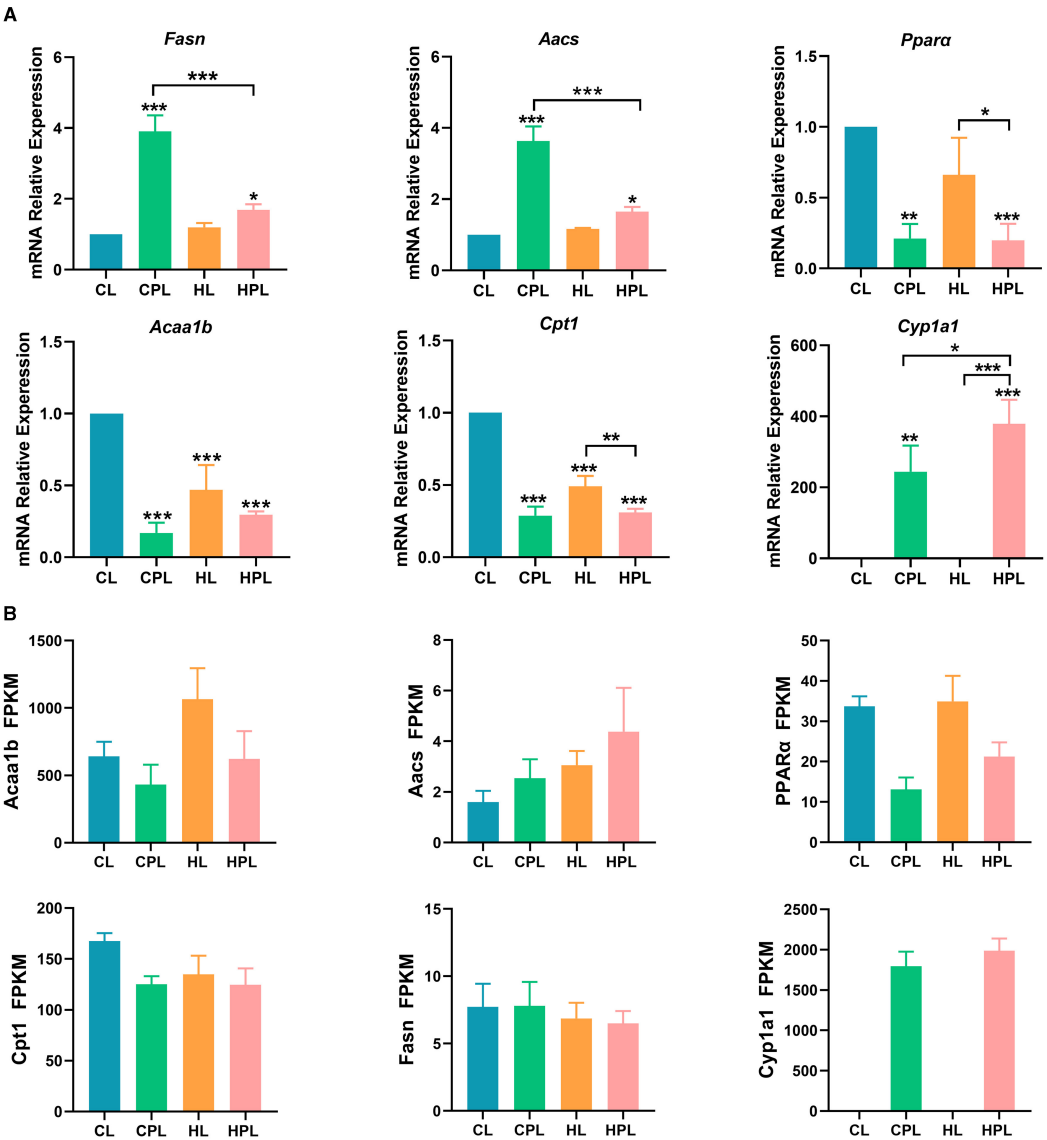
PCB169 exposure altered hepatic lipid metabolism-related gene expression. **(A)** Relative mRNA levels of lipogenesis genes and fatty acid oxidation genes. **(B)** The overall trends of RAN-seq and qPCR results were basically the same. * indicates *P < 0.05, **P < 0.01, ***P < 0.001 compared with the CL group.

We found that the expression of the fatty acid synthesis gene *Fasn* was significantly increased by 2.90-fold (*P* < 0.001) and 0.69-fold (*P* < 0.05) in the CPL and HPL groups, respectively, compared with the CL group (Figure 7A). *Fasn* gene expression in the HPL group was reduced by 56.6% (*P* < 0.001) compared with the CPL group. The expression of *Aacs*, a key gene regulating fatty acid synthesis, was significantly increased by 2.63-fold (*P* < 0.001) and 0.65-fold (*P* < 0.05) in the CPL and HPL groups compared with the CL group (Figure 7A). In addition, *Aacs* gene expression in the HPL group was reduced by 54.6% (*P* < 0.001) compared with the CPL group (Figure 7A).

The expression of *Ppar*α and *Acaalb*, key enzymes in fatty acid β-oxidation, was significantly lower in the CPL, HL and HPL groups compared with the CL group. Compared with the CL group, *Ppar*α gene expression was significantly reduced by 78.9% (*P* < 0.01) and 80.2% (*P* < 0.001) in the CPL and HPL groups, respectively (Figure 7A). *Ppar*α gene expression was reduced by 70.1% (*P* < 0.05) in the HPL and HL groups, respectively (Figure 7A). *Acaalb* gene expression was significantly reduced by 83.1% (*P* < 0.001), 53.1% (*P* < 0.001), and 70.5% (*P* < 0.001) in the CPL, HL, and HPL groups, respectively (Figure 7A). In comparison with the CL group, *Cpt1* is the rate-limiting enzyme in fatty acid β-oxidation, and *Cpt1* gene expression was significantly reduced by 71.1% (*P* < 0.001), 50.9% (*P* < 0.001), and 68.9% (*P* < 0.001) in the CPL, HL, and HPL groups, respectively, as compared with the CL group (Figure 7A). The HPL group showed a significant reduction in *Cpt1* gene expression by 36.8% (*P* < 0.01) compared with the HL group (Figure 7A). *Cyp1a1* gene is involved in fatty acid oxidation, and the expression of *Cyp1a1* gene was significantly increased 243.2-fold (*P* < 0.01) and 378.1-fold (*P* < 0.001) in the CPL and HPL groups, respectively, compared with the CL group (Figure 7A). In addition, *Cyp1a1* gene expression was increased 0.6-fold (*P* < 0.05) in the HPL group compared with the CPL group (Figure 7A). These gene expression data were consistent with the trend of the RNA-seq data (Figure 7B), and demonstrated that PCB169 promoted hepatic lipogenesis and inhibited lipid elimination in mice, which is consistent with the significant accumulation of lipids in mouse liver.

## 4 Discussion

**Figure 8:**
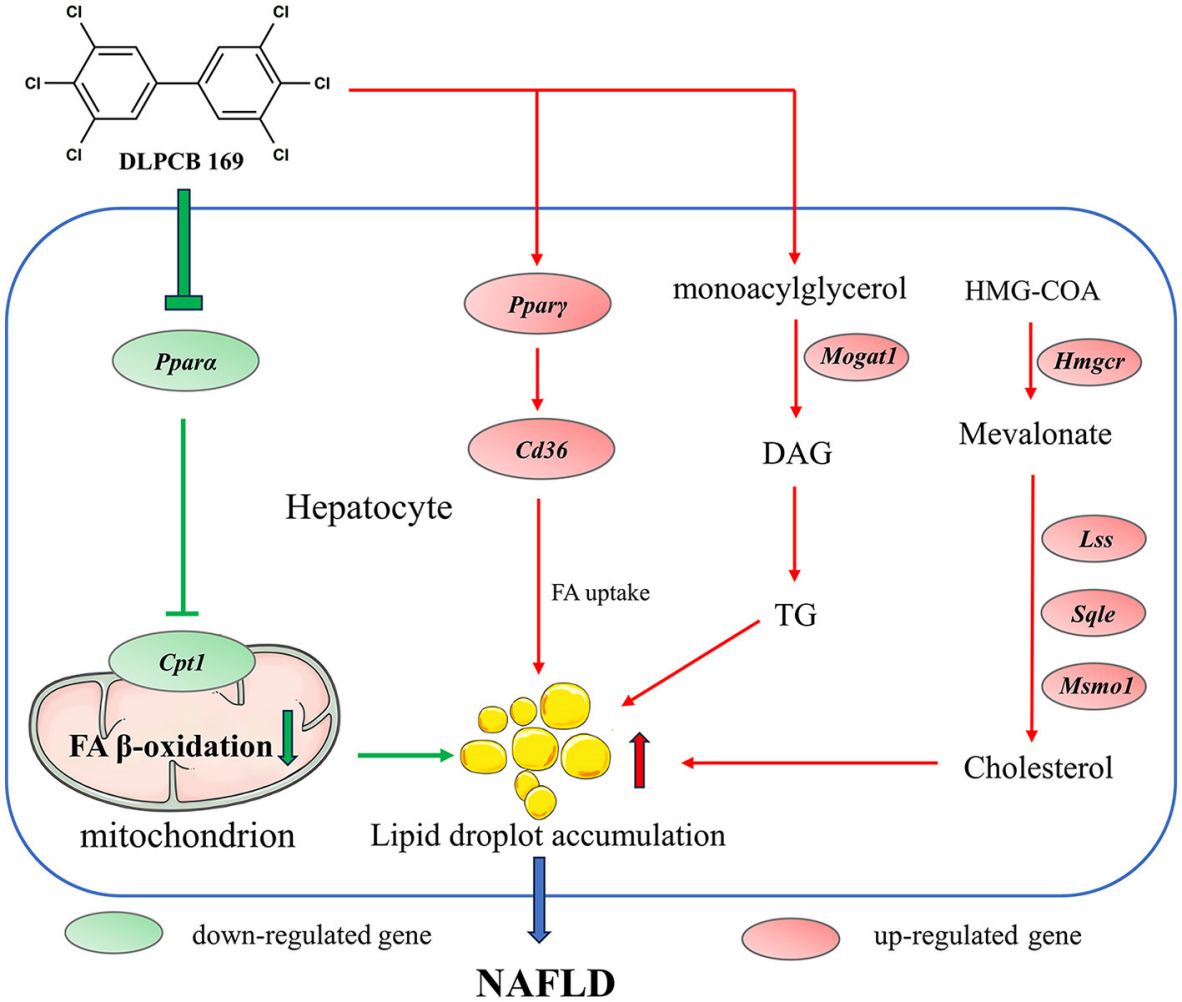
Schematic diagram of the molecular mechanism by which PCB169 exposure altered the lipid metabolic pathway in mouse liver. The expression of genes related to lipid accumulation and adipogenesis was significantly up-regulated by PCB169 through gavage treatment. PCB169 up-regulated the expression of *Ppar*γ and *Cd36* to promote fatty acid (FA) synthesis and endocytosis, and down-regulated the expression of *Ppar*α and *Cpt1* to reduce lipid oxidation. In addition, PCB169 increased TG and TC synthesis by regulating the expression of *Mogat1, Hmgcr, Lss, Sqle*, and *Msmo1*. Finally, PCB169 induces an increase in hepatic lipids that cannot be transported out of the liver, resulting in a vicious cycle of decreased body fat mass, weight loss, and positive feedback from the body to the liver to synthesize more fat. Red arrows indicate promotion and green arrows indicate inhibition.

The liver is a major metabolic organ in the animal body, regulating energy metabolism and lipid metabolism in the animal organism (6). In this study, we used mice to establish an *in vivo* model to study the role of PCB169 in the development of NAFLD and to understand the toxicity and molecular mechanisms of PCB169 exposure. As a result of this study, Mice exposed to PCB169 exhibited notable weight loss and reductions in fat mass. Intriguingly, the livers of these mice demonstrated abnormal lipid accumulation. This observation suggests that exposure to PCB169 may have a toxic effect, potentially disrupting lipid metabolism processes in mice. Our data suggested that PCB169 exposure altered the expression of genes involved in lipid metabolism and significantly increased the lipid content of the liver, including triglycerides and cholesterol, but the pathway of lipid transport out of the liver was significantly inhibited, resulting in a decrease in the amount of body fat in the mice, which led to the retardation of their growth and a significant reduction in their body weight (Figure 8).

It has been shown that DLPCB exposure induces AhR receptor activation (26) and causes transcription of many downstream genes, especially *Cyp1a1* (27). PCB169 is one of the most toxic DLPCB contaminants, and in the present study, the expression of *Cyp1a1* was significantly induced in the livers of mice by gavage of PCB169. However, studies have reported that the mechanism of DLPCB-induced NAFLD is complex and is likely to vary among compounds (28). Peroxisome proliferator-activated receptors (PPARs), including PPARα, PPARβ, and PPARγ, are key receptors that regulate lipid and glucose metabolism (29). Research findings indicate that DLPCB causes steatosis through the up-regulation of PPARγ, which leads to an increase in lipid accumulation, and the down-regulation of PPARα, which decreases fatty acid oxidation (28, 30). In our study, we observed that PCB169 exposure significantly activated the PPAR signaling pathway, a nuclear receptor that regulates the transport, esterification, and oxidation of fatty acids and plays an important role in lipid metabolism (31). In this study, PCB169 exposure resulted in an up-regulation of *Ppar*γ gene expression, resulting in increased lipid accumulation. Additionally, it down-regulated *Ppar*α gene expression, thereby inhibiting the fatty acid β-oxidation pathway and reducing fatty acid oxidation, which is critical for inducing hepatic lipid accumulation and degeneration. The present study is different from the mechanism of hepatic steatosis induced by PCB156 exposure. At the initial stage of PCB156 exposure, hepatic synthesis of fat was increased, and lipids were transported to the extrahepatic area into the bloodstream, and then transported to various parts of the body for deposition via apolipoproteins, and the body fat content was increased, and the mice were abnormally obese. With the prolongation of exposure time, PCB156 still induced an increase in hepatic lipids. However, this triggered a negative feedback mechanism in the liver, inhibiting fatty acid β-oxidation. This abnormal increase in hepatic lipid content ultimately led to the development of NAFLD (21). The differences in toxicity mechanisms between PCB156 and PCB169 exposure could potentially be attributed to their distinct chemical structures. In a previous study, PCB126 (2.45 mg/kg bw) exposed to mice caused hepatic steatosis (32). Decreased expression of Pparα and *Cpt1a* genes involved in fatty acid oxidation, and increased expression of *Cd36* genes involved in fatty acid transport in the liver imply impaired lipid oxidation and increased lipid uptake, which mechanistically explains the hepatic fat accumulation observed in the PCB126 exposed group (32). The findings of this study are consistent with those reported by PCB126. In previous studies, TCDD increased fatty acid uptake, inhibited β oxidation, reduced lipid effluence, and promoted hepatic steatosis in mice (33). In addition, exposure to TCDD 5 μg/kg TCDD for 3 weeks induced lipid deposition in the liver of C57BL/6J mice, and the level of CD36 protein was also increased (34). Previous studies have demonstrated that the interaction between AhR and PPAR pathways promotes *Cd36* gene expression (35), which was also found to be up-regulated in the present study. These results suggest that the AhR and PPAR pathways play an important role in the mechanism of PCB169-induced hepatic steatosis. In addition, the expression of *Fasn* and *Aacs*, genes that regulate lipid synthesis, was significantly up-regulated, and the expression of *Acaa1b* and β-oxidation rate-limiting enzyme *Cpt1* was significantly down-regulated, promoting lipid accumulation in the liver. In the present study, the changes in body weight and lipid metabolism gene expression were greater in PCB169 mice exposed to the control diet group than in mice exposed to the high-fat diet, and we hypothesized that the detrimental effects of low body fat content in mice might be ameliorated by dietary fat. Furthermore, our study revealed that PCB169 exposure significantly impacted various metabolic pathways such as Retinol metabolism, Metabolism of xenobiotics by cytochrome P450, Glutathione metabolism, Arachidonic acid metabolism, Steroid hormone biosynthesis, AGE-RAGE signaling pathway in diabetic complications, Fluid shear stress and atherosclerosis, Chemical carcinogenesis, Pathways in cancer, among others. Based on these findings, we hypothesized that PCB169 exposure could lead to lipid metabolism disorders, which promoted the development of NAFLD and further progressed to more serious diseases such as diabetes, atherosclerosis, and cancer. This finding provides new insights into the health effects of PCB169 exposure.

Studies have shown that PCB exposure interferes with cholesterol homeostasis and induces the development of NAFLD in mice (36). Increased cholesterol synthesis in the liver may affect membrane protein function and membrane fluidity, leading to the progression of NAFLD to more severe hepatitis and liver fibrosis diseases (37). In a previous study, hepatic TC levels were significantly increased in C57BL/6 mice exposed to PCB126 (38). Similarly, C57BL/6 mice exposed to PCB77 for 6 weeks and PCB156 for 12 weeks had increased hepatic TC content, respectively (21, 30). In our study, an increase in hepatic TC content due to PCB169 exposure was also observed, and the TC content in the HPL group was significantly increased by 1.38-fold (*P* < 0.01) compared with that in the CL group, but the elevation was not as significant as compared with the other groups. We hypothesized that the difference in results between PCB169 and other DLPCBs might be due to the different structures of DLPCBs. In addition, we found that PCB169 exposure upregulated the expression of cholesterol synthesis genes *Hmgcr, Sqle, Lss*, and *Msmol* thereby promoting cholesterol synthesis in the liver. We hypothesize that dysregulation of cholesterol homeostasis is an important process in PCB169 exposure-induced disorders of hepatic lipid metabolism. The accumulation of lipid droplets in hepatocytes is a typical pathological feature of NAFLD (39). In our study, PCB169 exposure induced a large number of lipid drop vacuoles in the livers of mice on a high-fat diet, with more severe hepatic steatosis and inflammatory damage, consistent with previous reports (20, 21). Interestingly, PCB169 exposure induced large fat drop vacuoles in the liver of mice with normal diet, which may be caused by metabolic disorders due to changes in liver gene expression. Furthermore, our study demonstrated that lower exposure doses exhibited opposite effects on increasing body weight compared to high doses of DLPCB exposure *in vivo*. The potential limitation of this study is that the experimental animals are only male mice, and whether female mice have different results due to gender differences needs further research.

## 5 Conclusion

Our study found that exposure to the persistent environmental pollutant PCB169 resulted in weight loss, significantly altered hepatic lipid metabolic pathways and lipid levels, and exacerbated liver injury in both normal and high-fat diet-induced mouse models. Lipid accumulation was more pronounced in the livers of mice co-exposed to PCB169 with a high-fat diet compared to the control diet. Analysis of RNA-seq and qPCR results showed that PCB169 promotes the development of NAFLD due to a significant increase in hepatic lipid synthesis, a significant decrease in lipid oxidation, and an inability to transport lipids to various parts of the body for deposition, resulting in an abnormal accumulation of lipids in the liver. Our study lays the foundation for understanding the toxic effects of PCB169 exposure in liver diseases and provides a theoretical basis for studying PCB169-induced NAFLD development.

## Data availability statement

The datasets presented in this study can be found in online repositories. The names of the repository/repositories and accession number(s) can be found below: https://www.ncbi.nlm.nih.gov/, PRJNA1041304.

## Ethics statement

All animal experiments were conducted in accordance with the guidelines for the care and use of laboratory animals of Guangdong Ocean University and approved by the Animal Ethics Committee of Guangdong Ocean University (Approval No. 2019090504) in an effort to minimize animal suffering. The study was conducted in accordance with the local legislation and institutional requirements.

## Author contributions

YW: Data curation, Investigation, Methodology, Writing – original draft. GZ: Conceptualization, Methodology, Writing – review & editing. GL: Conceptualization, Methodology, Writing – review & editing. WW: Conceptualization, Methodology, Writing – review & editing. LS: Methodology, Supervision, Writing – review & editing. HL: Methodology, Supervision, Writing – review & editing. JC: Conceptualization, Data curation, Funding acquisition, Methodology, Supervision, Writing – review & editing. DK: Conceptualization, Data curation, Funding acquisition, Methodology, Supervision, Writing – review & editing.

## Supplementary material

Supplementary Table 1 The ingredients of diet.

Supplementary Table 2 Primer sequences used for qPCR validation of genes.

Supplementary Table 3 Up-regulated and down-regulated DEGs in four comparison groups.

Supplementary Table 4 The specific and common DEGs in the four comparisons.

Supplementary Table 5 The DEGs used in hierarchical cluster analysis.

Supplementary Table 6 Summary of GO functional annotations for DEGs in the four comparison groups.

Supplementary Table 7 Summary of KEGG enrichment results for DEGs in the four comparison groups.

## References

[1] Powell EE Wong VW Rinella M. Non-alcoholic fatty liver disease. Lancet. (2021) 397:2212–24. 10.1016/S0140-6736(20)32511-3 33894145

[2] Sinn DH Kang D Choi SC Hong YS Zhao D Guallar E. Nonalcoholic fatty liver disease without metabolic-associated fatty liver disease and the risk of metabolic syndrome. Clin Gastroenterol Hepatol. (2023) 21:1873–80. 10.1016/j.cgh.2022.09.014 36152895

[3] Tanase DM Gosav EM Costea CF Ciocoiu M Lacatusu CM Maranduca MA. The intricate relationship between type 2 diabetes mellitus (T2DM), insulin resistance (IR), and nonalcoholic fatty liver disease (NAFLD). J Diabetes Res. (2020) 2020:3920196. 10.1155/2020/3920196 32832560 PMC7424491

[4] Zhao YC Zhao GJ Chen Z She ZG Cai J Li H. Nonalcoholic fatty liver disease: an emerging driver of hypertension. Hypertension. (2020) 75:275–84. 10.1161/HYPERTENSIONAHA.119.13419 31865799

[5] Lim JS Son HK Park SK Jacobs DR Lee DH. Inverse associations between long-term weight change and serum concentrations of persistent organic pollutants. Int J Obes (Lond). (2011) 35:744–7. 10.1038/ijo.2010.188 20820170

[6] Zheng S Yang Y Wen C Liu W Cao L Feng X. Effects of environmental contaminants in water resources on nonalcoholic fatty liver disease. Environ Int. (2021) 154:106555. 10.1016/j.envint.2021.106555 33857709

[7] Ruan J Guo J Huang Y Mao Y Yang Z Zuo Z. Adolescent exposure to environmental level of PCBs (Aroclor 1254) induces non-alcoholic fatty liver disease in male mice. Environ Res. (2020) 181:108909. 10.1016/j.envres.2019.108909 31776016

[8] Zhao D Zhang P Ge L Zheng GJ Wang X Liu W. The legacy of organochlorinated pesticides (OCPs), polycyclic aromatic hydrocarbons (PAHs) and polychlorinated biphenyls (PCBs) in Chinese coastal seawater monitored by semi-permeable membrane devices (SPMDs). Mar Pollut Bull. (2018) 137:222–30. 10.1016/j.marpolbul.2018.10.004 30503428

[9] Cao X Lu R Xu Q Zheng X Zeng Y Mai B. Distinct biomagnification of chlorinated persistent organic pollutants in adjacent aquatic and terrestrial food webs. Environ Pollut. (2023) 317:120841. 10.1016/j.envpol.2022.120841 36493935

[10] Ngoubeyou PSK Wolkersdorfer C Ndibewu PP Augustyn W. Toxicity of polychlorinated biphenyls in aquatic environments - a review. Aquat Toxicol. (2022) 251:106284. 10.1016/j.aquatox.2022.106284 36087490

[11] Giesy JP Kannan K. Dioxin-like and non-dioxin-like toxic effects of polychlorinated biphenyls (PCBs): implications for risk assessment. Crit Rev Toxicol. (1998) 28:511–69. 10.1080/10408449891344263 9861526

[12] Kannan VM Gopikrishna VG Saritha VK Krishnan KP Mohan M. PCDD/Fs, dioxin-like, and non-dioxin like PCBs in the sediments of high Arctic fjords, Svalbard. Mar Pollut Bull. (2022) 174:113277. 10.1016/j.marpolbul.2021.113277 34995883

[13] Terzaghi E Zanardini E Morosini C Raspa G Borin S Mapelli F. Rhizoremediation half-lives of PCBs: Role of congener composition, organic carbon forms, bioavailability, microbial activity, plant species and soil conditions, on the prediction of fate and persistence in soil. Sci Total Environ. (2018) 612:544–60. 10.1016/j.scitotenv.2017.08.189 28865272

[14] Fromme H Hilger B Albrecht M Gries W Leng G Völkel W. Occurrence of chlorinated and brominated dioxins/furans, PCBs, and brominated flame retardants in blood of German adults. Int J Hyg Environ Health. (2016) 219:380–8. 10.1016/j.ijheh.2016.03.003 27067547

[15] Aravind Kumar J Krithiga T Sathish S Renita AA Prabu D Lokesh S. Persistent organic pollutants in water resources: fate, occurrence, characterization and risk analysis. Sci Total Environ. (2022) 831:154808. 10.1016/j.scitotenv.2022.154808 35341870

[16] Montano L Pironti C Pinto G Ricciardi M Buono A Brogna C. Polychlorinated Biphenyls (PCBs) in the environment: occupational and exposure events, effects on human health and fertility. Toxics. (2022) 10:365. 10.3390/toxics10070365 35878270 PMC9323099

[17] Barone G Storelli A Busco A Mallamaci R Storelli MM. Polychlorinated dioxins, furans (PCDD/Fs) and dioxin-like polychlorinated biphenyls (DLPCBs) in food from Italy: Estimates of dietaryintake and assessment. J Food Sci. (2021) 86:4741–53. 10.1111/1750-3841.15901 34494668 PMC9293089

[18] Gao Q Ben Y Dong Z Hu J. Age-dependent human elimination half-lives of dioxin-like polychlorinated biphenyls derived from biomonitoring data in the general population. Chemosphere. (2019) 222:541–8. 10.1016/j.chemosphere.2019.01.168 30721813

[19] Boucher MP Lefebvre C Chapados NA. The effects of PCB126 on intra-hepatic mechanisms associated with non alcoholic fatty liver disease. J Diabetes Metab Disord. (2015) 14:88. 10.1186/s40200-015-0218-2 26693162 PMC4676123

[20] Wahlang B Falkner KC Gregory B Ansert D Young D Conklin DJ. Polychlorinated biphenyl 153 is a diet-dependent obesogen that worsens nonalcoholic fatty liver disease in male C57BL6/J mice. J Nutr Biochem. (2013) 24:1587–95. 10.1016/j.jnutbio.2013.01.009 23618531 PMC3743953

[21] Shan Q Chen N Liu W Qu F Chen A. Exposure to 2,3,3′,4,4′,5-hexachlorobiphenyl promotes nonalcoholic fatty liver disease development in C57BL/6 mice. Environ Pollut. (2020) 263:114563. 10.1016/j.envpol.2020.114563 32304952

[22] Wahlang B Song M Beier JI Cameron Falkner K Al-Eryani L Clair HB. Evaluation of Aroclor 1260 exposure in a mouse model of diet-induced obesity and non-alcoholic fatty liver disease. Toxicol Appl Pharmacol. (2014) 279:380–90. 10.1016/j.taap.2014.06.019 24998970 PMC4225625

[23] Arsenescu V Arsenescu RI King V Swanson H Cassis LA. Polychlorinated biphenyl-77 induces adipocyte differentiation and proinflammatory adipokines and promotes obesity and atherosclerosis. Environ Health Perspect. (2008) 116:761–8. 10.1289/ehp.10554 18560532 PMC2430232

[24] DeVito M Bokkers B van Duursen MBM van Ede K Feeley M Antunes Fernandes Gáspár E. The 2022 world health organization reevaluation of human and mammalian toxic equivalency factors for polychlorinated dioxins, dibenzofurans and biphenyls. Regul Toxicol Pharmacol. (2024) 146:105525. 10.1016/j.yrtph.2023.105525 37972849 PMC10870838

[25] Love MI Huber W Anders S. Moderated estimation of fold change and dispersion for RNA-seq data with DESeq2. Genome Biol. (2014) 15:550. 10.1186/s13059-014-0550-8 25516281 PMC4302049

[26] Eti NA Flor S Iqbal K Scott RL Klenov VE Gibson-Corley KN. PCB126 induced toxic actions on liver energy metabolism is mediated by AhR in rats. Toxicology. (2022) 466:153054. 10.1016/j.tox.2021.153054 34848246 PMC8748418

[27] Du F Zhao T Ji HC Luo YB Wang F Mao GH. Dioxin-like (DL-) polychlorinated biphenyls induced immunotoxicity through apoptosis in mice splenocytes via the AhR mediated mitochondria dependent signaling pathways. Food Chem Toxicol. (2019) 134:110803. 10.1016/j.fct.2019.110803 31563530

[28] Wahlang B Hardesty JE Jin J Falkner KC Cave MC. Polychlorinated biphenyls and nonalcoholic fatty liver disease. Curr Opin Toxicol. (2019) 14:21–8. 10.1016/j.cotox.2019.06.001 34485777 PMC8412140

[29] Ougerat A Montagner A Loiseau N Guillou H Wahli W. Peroxisome proliferator-activated receptors and their novel ligands as candidates for the treatment of non-alcoholic fatty liver disease. Cells. (2020) 9:1638. 10.3390/cells9071638 32650421 PMC7408116

[30] Chi Y Wang H Lin Y Lu Y Huang Q Ye G. Gut microbiota characterization and lipid metabolism disorder found in PCB77-treated female mice. Toxicology. (2019) 420:11–20. 10.1016/j.tox.2019.03.011 30935970

[31] Qiu YY Zhang J Zeng FY Zhu YZ. Roles of the peroxisome proliferator-activated receptors (PPARs) in the pathogenesis of nonalcoholic fatty liver disease (NAFLD). Pharmacol Res. (2023) 192:106786. 10.1016/j.phrs.2023.106786 37146924

[32] Wahlang B Perkins JT Petriello MC Hoffman JB Stromberg AJ Hennig B. compromised liver alters polychlorinated biphenyl-mediated toxicity. Toxicology. (2017) 380:11–22. 10.1016/j.tox.2017.02.001 28163111 PMC5374277

[33] Nault R Fader KA Lydic TA Zacharewski TR. Lipidomic evaluation of aryl hydrocarbon receptor-mediated hepatic steatosis in male and female mice elicited by 2,3,7,8-tetrachlorodibenzo-p-dioxin. Chem Res Toxicol. (2017) 30:1060–75. 10.1021/acs.chemrestox.6b00430 28238261 PMC5896278

[34] Cong Y Hong Y Wang D Cheng P Wang Z Xing C. 2,3,7,8-Tetrachlorodibenzo-p-dioxin induces liver lipid metabolism disorder via the ROS/AMPK/CD36 signaling pathway. Toxicol Sci. (2023) 191:276–84. 10.1093/toxsci/kfac133 36534932

[35] Kawano Y Nishiumi S Tanaka S Nobutani K Miki A Yano Y. Activation of the aryl hydrocarbon receptor induces hepatic steatosis via the upregulation of fatty acid transport. Arch Biochem Biophys. (2010) 504:221–7. 10.1016/j.abb.2010.09.001 20831858

[36] Shen X Chen Y Zhang J Yan X Liu W Guo Y. Low-dose PCB126 compromises circadian rhythms associated with disordered glucose and lipid metabolism in mice. Environ Int. (2019) 128:146–57. 10.1016/j.envint.2019.04.058 31055201

[37] Ioannou GN. The role of cholesterol in the pathogenesis of NASH. Trends Endocrinol Metab. (2016) 27:84–95. 10.1016/j.tem.2015.11.008 26703097

[38] Chi Y Lin Y Lu Y Huang Q Ye G Dong S. Gut microbiota dysbiosis correlates with a low-dose PCB126-induced dyslipidemia and non-alcoholic fatty liver disease. Sci Total Environ. (2019) 653:274–82. 10.1016/j.scitotenv.2018.10.387 30412872

[39] Sztalryd C Brasaemle DL. The perilipin family of lipid droplet proteins: gatekeepers of intracellular lipolysis. Biochim Biophys Acta Mol Cell Biol Lipids. (2017) 1862:1221–32. 10.1016/j.bbalip.2017.07.009 28754637 PMC5595658

